# Morphological and Genetic Analysis of the *Acerentomon doderoi* Group (Protura: Acerentomidae) with Description of *A*. *christiani* sp. nov

**DOI:** 10.1371/journal.pone.0148033

**Published:** 2016-04-13

**Authors:** Julia Shrubovych, Daniela Bartel, Nikolaus Urban Szucsich, Monika Carol Resch, Günther Pass

**Affiliations:** 1 Institute of Systematics and Evolution of Animals, Polish Academy of Sciences, Kraków, Poland; 2 Department of Integrative Zoology, University of Vienna, Vienna, Austria; 3 3rd Department of Zoology, Museum of Natural History Vienna, Vienna, Austria; Saint Mary's University, CANADA

## Abstract

*Acerentomon christiani* sp. nov. is described from Vienna, Austria. The new species is a member of the “*doderoi*” group, characterized by the presence of seta *x* on tergite VII. It is most similar to *A*. *gallicum*, *A*. *brevisetosum* and *A*. *tenuisetosum*, but differs from these species in the length of foretarsal sensillum *c* and certain other chaetotaxic measurements and indices. In addition to the morphological description, the DNA barcoding region of the mitochondrial cytochrome c oxidase subunit 1 gene (COI) and the 28S ribosomal RNA of the new species are provided. The morphological characters and the barcode of the new species are discussed in comparison to those of other *Acerentomon* species. An identification key to all known *Acerentomon* spp. of the “*doderoi*” group is given.

## Introduction

Until recently, the taxonomy of Protura was based exclusively on morphology. Many characters are inconspicuous and difficult to recognize, often making a reliable species determination problematic even for long-experienced experts (for review see [1]). However, it has been convincingly demonstrated [2] that molecular barcodes are an additional important and useful tool in the taxonomy of these minute soil arthropods. Use of barcodes has revealed that morphological taxonomy is reflected very closely by the molecular data. This result is of special importance since the reproductive biology of proturans remains enigmatic to date (for review see [3]). As a consequence it is not possible to check the described morphospecies from a biospecies perspective. Therefore, whenever possible each new species description of Protura should include a molecular characterization by barcodes as well as the description of morphological characters.

The genus *Acerentomon* Silvestri, 1907 presently comprises 38 species [1], [4], [5]), all of which have a West Palaearctic distribution (Europe and northern Africa). Most species are recorded solely from their type localities. Thirteen species of the genus *Acerentomon* have been reported from Austria ([6]). In soil samples from the Leopoldsberg (near Vienna) we found an undescribed species of *Acerentomon* together with *A*. *italicum* Nosek, 1969, both belonging to the “*doderoi*” group sensu Nosek (1973) [7]. The present paper contains a description of the new species from Austria, along with its barcoding sequence, and an identification key for all *Acerentomon* species of the *doderoi* group.

## Materials and Methods

### Ethics Statement

The species used in this study are neither CITES-species nor endangered species according to regional Red Lists. Our sampling permission was RU5-BE-939/001-2013.

### Nomenclature Acts

The electronic edition of this article conforms to the requirements of the amended International Code of Zoological Nomenclature, and hence the new names contained herein are available under that Code from the electronic edition of this article. This published work and the nomenclatural acts it contains have been registered in ZooBank, the online registration system for the ICZN. The ZooBank LSIDs (Life Science Identifiers) can be resolved and the associated information viewed through any standard web browser by appending the LSID to the prefix "http://zoobank.org/". The LSID for this publication is: urn:lsid:zoobank.org:pub: 1F2DDC05-5E40-4127-AB12-FC089F40EEAE. The electronic edition of this work was published in a journal with an ISSN, and has been archived and is available from the following digital repositories: PubMed Central, LOCKSS.

### Morphological Approach

All specimens were mounted on microscopic slides in Faure medium or Marc Andre II medium. Head setae are labeled as in [8]. Body chaetotaxy is given as in [9] with the following changes: setae A0 and P0 are labeled as *Ac* and *Pc*; chaetotaxy of tergite VIII is labeled as in [10]. Terminology for body porotaxy follows [11] and [12]. Abbreviations used in the description are as follows: Abd.–abdominal segments, Th.–thoracic segments, *psm* = posterosubmedial, *psl* = posterosublateral, *al* = anterolateral, *sam* = sternal anteromedial, *sc* = sternal central, *spm* = sternal posteromedial, *spsm* = sternal posterosubmedial, *spsl* = sternal posterosublateral pore.

### Molecular Approach

To obtain COI and 28S (region D2–D3) sequences, genomic DNA was extracted from complete animals applying a non-destructive extraction method (NDE) [13]. Protocols for DNA extraction, amplification, and sequencing of 41 published individuals from the genus *Acerentomon* are given in [2]. In the present study, DNA of 27 individuals was extracted by means of an NDE-method with the Blood & Tissue Kit (Qiagen). After DNA extraction the cuticle was transferred to 100% EtOH, before wholemounts were prepared. Thermocycler profiles differed for the amplification of the COI and 28S rDNA fragment: initial denaturation of 30 sec, 30 cycles of 1 min at 94°C, 1 min at 46°C (COI) / 48°C (28S rDNA) and 1 min (COI) / 1.5 min (28S rDNA) at 68°C and a final extension step for 5 min at 68°C.

Each PCR reaction consisted of 2 μl DNA, 5 μl PCR buffer (5x containing 18 mM MgCl_2_; BioLabs OneTaq), 0.7 μl dNTP (10 mM each; BioLabs Desoxynucleotide Solution Mix), 0.7 μl each primer (10 μM, VBC Biotech), 0.1 μl Polymerase (BioLabs OneTaq), 10.8 μl ddH_2_O. A second PCR-repeat, applying the same conditions, was used to increase the yield of DNA. PCR products were purified using GeneJET PCR Purification Kit (Thermo Scientific) and eluted in 20 μl with ddH_2_O. Sequencing reaction was performed at LGC Genomics, Germany. Different primer pairs were needed to sucessfully amplify the COI and 28S rDNA fragments (Table 1). In this study we successfully sequenced 13 sequences of the COI, and 27 sequences of the 28S rDNA. All sequences from the study of [2] are deposited at the Barcode of Life Data Systems (BOLD) under the project name PROTAT. New sequences are deposited at BOLD under the project name PROTA (Table 2). The entire dataset of the manuscript can be downloaded at: http://www.boldsystems.org/index.php/MAS_Management_OpenDataSet?datasetcode=DS-2016ADOD

**Table 1:** List of primers used in the present study.

Locus	primer cocktail	Primer name	direction	Sequences (3’-5’)
COI	no	LCO1490	forward	ggtcaacaaatcataaagatattgg
	no	LepF1	forward	attcaaccaatcataaagatattgg
	no	DiplR1	reverse	gcaataattatdgtdgctgc
	yes	GlomF1	forward	Prot F4/1 + CamF1 (1:1)
	no	ProtF4/1	forward	ctcractaaccataargatatcgg
	no	CamF1	forward	ctcractaaccataargatattgg
	yes	GlomR1	reverse	JapR1 + CamR1 + ProtR1 (1:1:1)
	yes	LCO, LepF1	forward	LCO + LepF1 (1:1)
	no	JapR1	reverse	tayacttcdgggtgbccaaagaatc
	no	CamR1	reverse	taaacttcdggrtgdccaaaaaatc
	no	ProtR1	reverse	taaacttcwggrtgsccaaaraatc
28S rDNA	no	D2a	forward	gatagcgaacaagtacc
	no	D2aProt	forward	gtaccgcgagggaaagttg
	no	D3b	reverse	tccggaaggaaccagctacta
	no	D3bProt2	reverse	gaaagactaatcgaaccatc
	no	D3bProt2	reverse	ctcractaaccataargatatcgg

**Table 2:** List of investigated proturans.

Ind.ID	Species name	Sampling location	COI (BOLD)	28S (BOLD)
HP006	Acerentomon cf. italicum	Leopoldsberg	-	PROAT091-13
HP010	Acerentomon sp.	Leopoldsberg	-	PROAT092-13
HP013	Acerentomon sp.	Twimberger Graben	PROAT005-12	PROAT005-12
HP014	Acerentomon sp.	Twimberger Graben	PROAT006-12	PROAT006-12
HP042	Acerentomon maius	Twimberger Graben	PROAT001-12	PROAT001-12
HP044	Acerentomon maius	Twimberger Graben	PROAT015-12	PROAT015-12
HP045	Acerentomon maius	Twimberger Graben	PROAT016-12	PROAT016-12
HP046	Acerentomon maius	Twimberger Graben	PROAT017-12	PROAT017-12
HP047	Acerentomon maius	Twimberger Graben	-	PROAT096-13
HP052	Acerentomon maius	Twimberger Graben	PROAT018-12	PROAT018-12
HP058	Acerentomon maius	Twimberger Graben	PROAT019-12	PROAT019-12
HP059	Acerentomon maius	Twimberger Graben	PROAT020-12	PROAT020-12
HP061	Acerentomon carpaticum	Twimberger Graben	PROAT022-12	PROAT022-12
HP067	Acerentomon cf. maius	Twimberger Graben	PROAT024-12	PROAT024-12
HP075	Acerentomon christiani sp. nov.	Leopoldsberg	PROAT025-12	-
HP076	Acerentomon christiani sp. nov.	Leopoldsberg	PROAT026-12	PROAT026-12
HP077	Acerentomon christiani sp. nov.	Leopoldsberg	PROAT027-12	PROAT027-12
HP079	Acerentomon christiani sp. nov.	Leopoldsberg	PROAT029-12	-
HP085	Acerentomon christiani sp. nov.	Leopoldsberg	PROAT030-12	PROAT030-12
HP086	Acerentomon christiani sp. nov.	Leopoldsberg	PROAT031-12	PROAT031-12
HP087	Acerentomon christiani sp. nov.	Leopoldsberg	PROAT032-12	PROAT032-12
HP089	Acerentomon christiani sp. nov.	Leopoldsberg	PROAT033-12	PROAT033-12
HP090	Acerentomon christiani sp. nov.	Leopoldsberg	PROAT034-12	PROAT034-12
HP096	Acerentomon christiani sp. nov.	Leopoldsberg	PROAT035-12	PROAT035-12
HP099	Acerentomon christiani sp. nov.	Leopoldsberg	-	PROAT100-13
HP100	Acerentomon christiani sp. nov.	Leopoldsberg	PROAT036-12	PROAT036-12
HP101	Acerentomon christiani sp. nov.	Leopoldsberg	PROAT037-12	PROAT037-12
HP102	Acerentomon christiani sp. nov.	Leopoldsberg	PROAT038-12	PROAT038-12
HP103	Acerentomon christiani sp. nov.	Leopoldsberg	PROAT039-12	-
HP106	Acerentomon christiani sp. nov.	Leopoldsberg	PROAT040-12	PROAT040-12
HP107	Acerentomon christiani sp. nov.	Leopoldsberg	PROAT041-12	PROAT041-12
HP108	Acerentomon christiani sp. nov.	Leopoldsberg	PROAT042-12	PROAT042-12
HP132	Acerentomon italicum	Leopoldsberg	PROAT052-12	PROAT052-12
HP135	Acerentomon christiani sp. nov.	Leopoldsberg	PROAT054-12	PROAT054-12
HP136	Acerentomon christiani sp. nov.	Leopoldsberg	PROAT055-12	PROAT055-12
HP144	Acerentomon christiani sp. nov.	Leopoldsberg	PROAT063-12	PROAT063-12
HP145	Acerentomon christiani sp. nov.	Leopoldsberg	PROAT064-12	PROAT064-12
HP146	Acerentomon christiani sp. nov.	Leopoldsberg	PROAT065-12	PROAT065-12
HP147	Acerentomon christiani sp. nov.	Leopoldsberg	PROAT066-12	PROAT066-12
HP150	Acerentomon christiani sp. nov.	Leopoldsberg	PROAT069-12	PROAT069-12
HP152	Acerentomon christiani sp. nov.	Leopoldsberg	PROAT071-12	PROAT071-12
1558_PROTA	Acerentomon dispar	Trebesiner Weg	PROTA001-15	PROTA001-15
1561_PROTA	Acerentomon dispar	Trebesiner Weg	PROTA002-15	PROTA002-15
1562_PROTA	Acerentomon carpaticum	Slovakia	PROTA003-15	PROTA003-15
1563_PROTA	Acerentomon carpaticum	Slovakia	PROTA004-15	PROTA004-15
1564_PROTA	Acerentomon carpaticum	Slovakia	-	PROTA005-15
1575_PROTA	Acerentomon carpaticum	Slovakia	PROTA006-15	PROTA006-15
1576_PROTA	Acerentomon carpaticum	Slovakia	PROTA007-15	PROTA007-15
2844_PROTA	Acerentomon dispar	Leithagebirge	PROTA008-15	PROTA008-15
2845_PROTA	Acerentomon dispar	Leithagebirge	PROTA009-15	PROTA009-15
3357_PROTA	Acerentomon dispar	Bachwinkl	-	PROTA010-15
3359_PROTA	Acerentomon dispar	Bachwinkl	-	PROTA011-15
3360_PROTA	Acerentomon dispar	Bachwinkl	-	PROTA012-15
3361_PROTA	Acerentomon dispar	Bachwinkl	PROTA013-15	PROTA013-15
3362_PROTA	Acerentomon dispar	Bachwinkl	-	PROTA014-15
3364_PROTA	Acerentomon dispar	Bachwinkl	-	PROTA015-15
3365_PROTA	Acerentomon dispar	Bachwinkl	-	PROTA016-15
3366_PROTA	Acerentomon dispar	Bachwinkl	-	PROTA017-15
3447_PROTA	Acerentomon dispar	Kremstal	-	PROTA018-15
3449_PROTA	Acerentomon dispar	Kremstal	-	PROTA019-15
3450_PROTA	Acerentomon dispar	Kremstal	-	PROTA020-15
3451_PROTA	Acerentomon dispar	Kremstal	PROTA021-15	PROTA021-15
3452_PROTA	Acerentomon dispar	Kremstal	-	PROTA022-15
3453_PROTA	Acerentomon dispar	Kremstal	PROTA023-15	PROTA023-15
3454_PROTA	Acerentomon dispar	Kremstal	PROTA024-15	PROTA024-15
3455_PROTA	Acerentomon dispar	Kremstal	PROTA025-15	PROTA025-15
3457_PROTA	Acerentomon dispar	Kremstal	-	PROTA026-15
3458_PROTA	Acerentomon dispar	Kremstal	-	PROTA027-15

Alignment and NJ tree based on K2P distances were performed using MUSCLE, as implemented in Mega v. 6.0 [14]. COI and 28S rDNA were analyzed separately. The reliability of trees was assessed with 1000 bootstrap replicates.

## Results

### Systematics of the Genus *Acerentomon*

Protura of the genus *Acerentomon* Silvestri, 1907 are characterized by the presence of three pairs of anterior setae on the mesonotum and four pairs of anterior setae on the metanotum, well developed maxillary palps with an apical tuft of setae and basal sensilla, a striate band with distinct striae, a claviform foretarsal sensillum *t1*, a leaf-like sensillum *t3*, a very long seta *δ4* which contrasts with the length of other *δ*-setae, and the absence of sensillum *b'*. The second and third abdominal legs usually carry two setae differing in length (apical seta half the length of subapical seta). The maxillary gland possesses a small calyx and a short distal part. The genus has been subdivided into four groups, which differ in the presence or absence of seta *x* on tergite VII, and seta *P1a* on sternite VIII ([7], [10]). However, a cladistic analysis performed on 36 *Acerentomon* species using 24 morphological characters supports a split into only two main groups: “*doderoi*” and “*aceris*” [15]. Of these the “*doderoi*” group is characterized by the presence of seta *x* on tergite VII, and comprises a total of 21 species. Two *Acerentomon* spp. described after the study can be assigned to the *aceris* group.

The new species described in this paper belongs to the “*doderoi*” group of *Acerentomon* species in the sense of [15] and is characterized by the presence of seta *x* on tergite VII and a pair of posterior setae *P1a* on sternite VIII, presence of seta *Pc* on tergite VII and sternite VII, absence of seta *P3a* on tergite VII, long mesonotal and metanotal setae *P1a* that are longer than *P1*, long foretarsal sensillum *c*, short and slender sensillum *b*, and broadened sensillum *a*. The new species is most similar to *A*. *gallicum* Ionescu, 1933 in chaetotaxy and measurements. A detailed description is given below.

### *Acerentomon christiani* sp. nov. Shrubovych & Resch

urn:lsid:zoobank.org:act:A1685124-1A18-4F26-925C-7E25FD8D7453

### Morphological description

**Fig 1:**
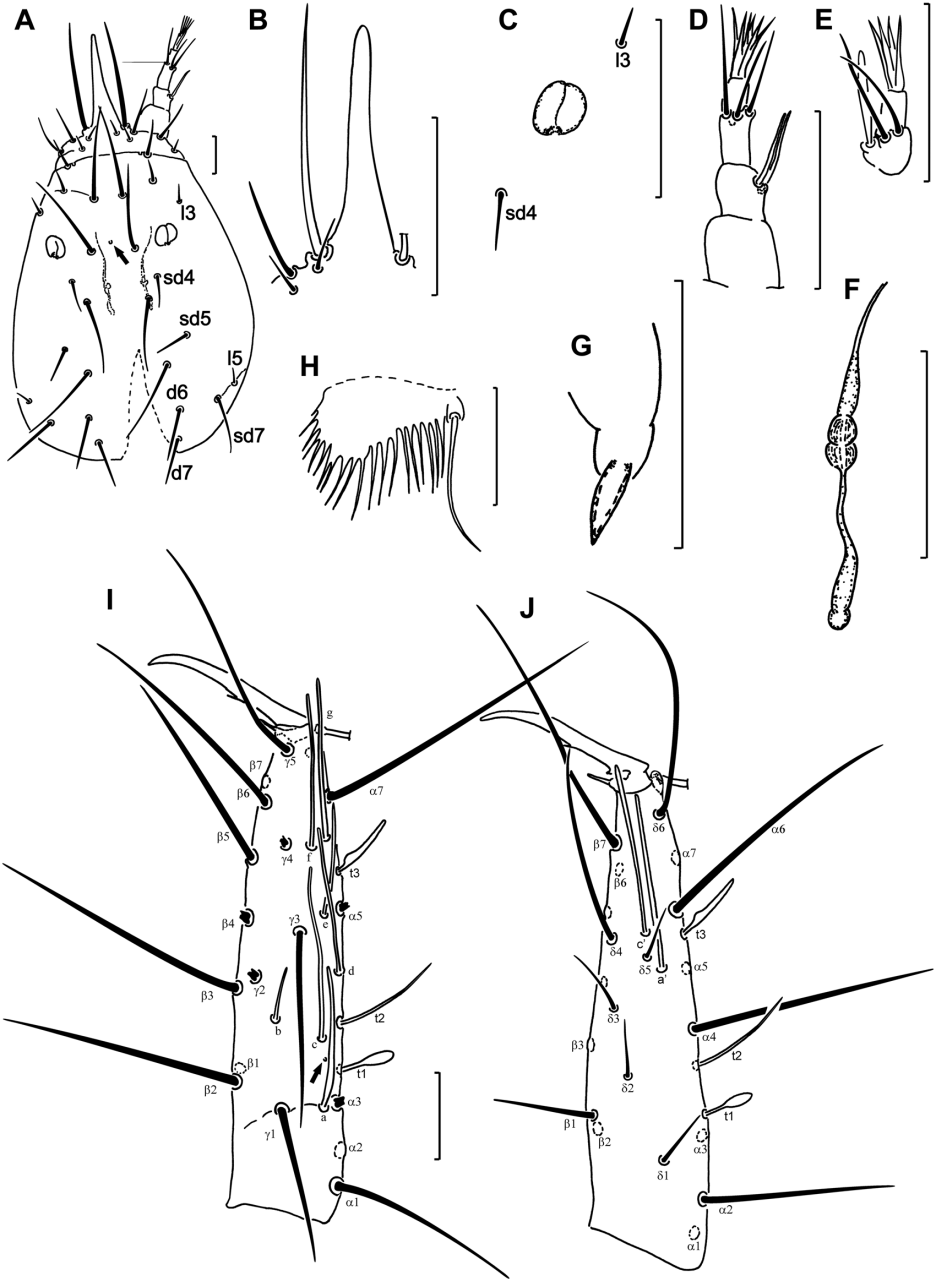
*Acerentomon christiani* sp. nov. (A) Head. (B) Rostrum. (C) Pseudoculus. (D) Maxillary palp. (E) Labial palp. (F) Maxillary gland. (G) Acrostylus. (H) Comb. (I) Foretarsus, exterior view. (J) Foretarsus, interior view. Arrows indicate pores. All figures are of holotype. Scale bars: 20 μm.

***Characters for diagnosis*:** Head setae long, setiform, not modified. Posterior margin of head with seta *d7* slightly shorter than seta *sd7*. Additional seta *d6* present (Fig 1A). Rostrum long, LR 3.4‒3.8 (Fig 1B). Pseudoculus longer than broad, PR 16‒17 (Fig 1C). Sensilla of maxillary palp nearly equal in length (Fig 1D). Labial palps well developed, with broad basal sensillum (Fig 1E). Canal of maxillary gland with distinct thickening in posterior part and simple posterior dilation, 1.4‒1.6 times the length of the pseudoculus, CF 10.0‒11.9 (Fig 1F).

**Fig 2:**
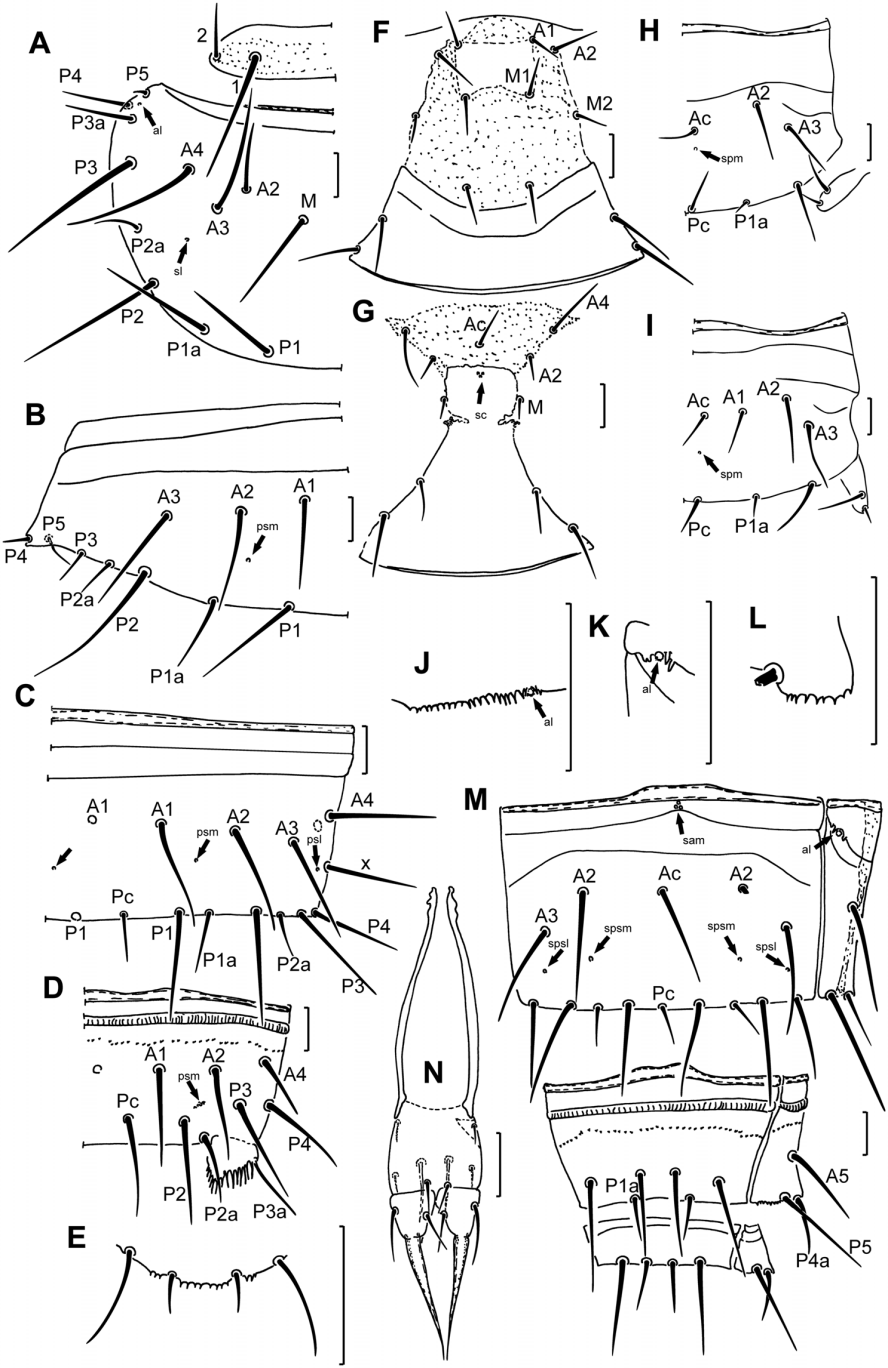
*Acerentomon christiani* sp. nov. (A) Pronotum and mesonotum (left side). (B) Tergite I (left side). (C) Tergite VII (right side). (D) Tergite VIII (right side). (E) Hind margin of sternite XII. (F) Prosternum. (G) Mesosternum. (H) Sternite II (right side). (I) Sternite III (right side). (J) Anterolateral structures on tergite VI. (K) Anterolateral structures on tergite VII. (L) Hind margin of laterotergite VIII. (M) Sternites VII–IX. (N) Male sguama genitalis. Arrows indicate pores (*al* = anterolateral, *sl* = sublateral, *psm* = posterosubmedial, *psl* = posterosublateral, *sam* = sternal anteromedial, *sc* = sternal central, *spm* = sternal posteromedial, *spsm* = sternal posterosubmedial, *spsl* = sternal posterosublateral pore). Figure N; paratype Cat. N 840+HP 136, other figures are of holotype. Scale bars: 20 μm.

Chaetotaxy formula given in Table 3. Setae on nota differing distinctly in length (Fig 2A). Length ratio of pronotal setae *1*:*2* as 2:1. Seta *M* on meso- and metanota longer than seta *A2*, which are approximately 50 and 30 μm, respectively. Accessory setae *P1a* and *P2a* setiform, differing in length; *P1a* slightly longer than *P1*, *P2a* one-third the length of *P1a* (Fig 2A). Setae *P3a* and *P4* subequal in length, short, setiform; *P5* a small sensillum. Length ratio of *P1*:*P1a*:*P2* on mesonotum as 0.9:1:1.4. Pronotum lacking pores. Mesonotum with 2+2 pores (*al*, *sl*) (Fig 2A). Metanotum with 1+1 *sl* pores. Prosternum with seta *M2*, meso- and metasterna without *A1* setae (Fig 2F and 2G). Setae *A2* and *M2* on prosternum and *A2* on meso- and metasterna setiform. Lateral margin of meso- and metasternum with distinct coxal incision (Fig 2G). Prosternum lacking pores; meso- and metasterna usually with three or four closely adjacent sternal central pores (*sc*), situated posterior to seta *Ac* (Fig 2G).

**Table 3:** Body chaetotaxy of *Acerentomon christiani* sp. nov.

	Dorsal		Ventral	
Segment	Formula	Setal composition	Formula	Setal composition
Th. I	4	1, 2	4+4	A1, 2, M1, 2
	4	1, 2	6	P1, 2, 3
Th. II	8	A2, 3, 4, M	5+2	Ac, 2, 4, M
	16	P1, 1a, 2, 2a, 3, 3a, 4, 5	4	P1, 3
Th. III	10	A2, 3, 4, 5, M	7+2	Ac, 2, 3, 4, M
	16	P1, 1a, 2, 2a, 3, 3a, 4, 5	4	P1, 3
Abd. I	6	A1, 2, 3	3	Ac, 2
	14	P1, 1a, 2, 2a, 3, 4, 5	4	P1, 1a
Abd. II	10	A1, 2, 3, 4, 5	5	Ac, 2, 3
	16	P1, 1a, 2, 2a, 3, 4, 4a, 5	5	Pc, 1a, 2
Abd. III	10	A1, 2, 3, 4, 5	7	Ac, 1, 2, 3
	16	P1, 1a, 2, 2a, 3, 4, 4a, 5	5	Pc, 1a, 2
Abd. IV-VI	10	A1, 2, 3, 4, 5	7	Ac, 1, 2, 3
	16	P1, 1a, 2, 2a, 3, 4, 4a, 5	8	P1, 1a, 2, 3
Abd. VII	12	A1, 2, 3, 4, 5, x	5	Ac, 2, 3
	17	Pc, 1, 1a, 2, 2a, 3, 4, 4a, 5	9	Pc, 1, 1a, 2, 3
Abd. VIII	8	A1, 2, 4, 5	4	1, 2
	16	Pc, P2, 2a, 3, 3a, 4, 4a, 5	2	1a
Abd. IX	14	1, 1a, 2, 2a, 3, 3a, 4	4	1, 2
Abd. X	10	1, 2, 2a, 3, 4	4	1, 2
Abd. XI	6	1, 3, 4	6	
Abd. XII	9		6	

Sensillum *t1* claviform; *t3* leaf-like; *a* sword-shaped; *b* slender, nearly setiform; other sensilla parallel-sided (Fig 1I and 1J). Sensillum *a* long, reaching base of sensillum *d*; *b* very short, its apex not reaching base of *γ3*; *c* very long, longer than *a* and three times longer than *b*, its base proximal to base of sensillum *b* and its apex reaching the base of *t3*; sensillum *e* located halfway between bases of sensilla *d* and *f* (Fig 1I). Sensilla *a’* and *c’* long. Sensillum *a’* located distal to level of *t2* insertion, close to the base of *c’* (Fig 1J. Seta *β1* setiform, longer than *δ1* setae, *δ4* setiform, very long in comparison with other *δ*-setae (Fig 1J). Claw long and slender, with small inner tooth. Empodial appendage short. Relative length of foretarsal sensilla: *t1* = *b* < *t3* < *t2* < *e* < *a* < *(c* = *f)* < *(d* = *g)* < *(a’* = *c’)*. BS 0.5; TR 3.0–3.1; EU 0.1–0.2. Pores present near base of sensilla *c* and *t3*.

Accessory setae on tergite I differing in length: seta *P1a* slightly shorter than *P1*, seta *P2a* one third length of *P2*, equal in length to *P3*, *P4* and *P5* setae (Fig 2B). Accessory setae on tergites II-VII setiform, nearly equal in length, about half the length of the principal setae (Fig 2C). Tergite VII with seta *Pc* and seta *x* (Fig 2D). Tergite I with 1+1 *psm* pores only (Fig 2B). Tergites II ‒VII with three pairs of pores (*psm*, *psl* and *al*) (Fig 2C and 2M). Tergites I–VII anteriorly with two parallel cuticular lines (Fig 2C and 2D). Pleural structures not developed on tergites I–V; on tergite VI 20–22 teeth present anterior to pore *al*, on tergite VII some distinct teeth near to pore *al* (Fig 2J and 2K).

Abdominal appendages with 4, 2, 2 setae. Apical seta of abdominal legs II and III less than half the length of subapical seta, 26 and 17 μm, respectively (Fig 2H and 2I). Accessory setae on sternites setiform, shorter than principal setae, seta *Pc* on sternite VII shorter than *P1a* setae (Fig 2M). Sternite I without pores, sternites II–V with single *spm* pore posterior to *Ac* (Fig 2H and 2I). Sternite VI and VII with two pairs of *spsm* and *spsl* pores and with a group of three sternal anteromedial pores (*sam*) anterior to *Ac* and above cuticular lines (Fig 2M). Sternites II–III anteriorly with a cuticular line (Fig 2H and 2I), sternites IV–VII with two parallel cuticular lines (Fig 2M).

Abdominal segment VIII with distinct striate band and with a regular row of small, scattered denticles anteriorly (Fig 2D and 2M). Comb VIII composed of 14‒16 slender, teeth of varying lengths (Fig 1H). Pore *psm* with several surrounding teeth, other pores absent (Fig 2D). Laterotergites VIII with row of granules in anterior part and with 8‒10 small teeth on posterior margin (Fig 2L). Pore *psm* with 1‒2 accompanying teeth. Setae 1a present on sternite VIII. Pores absent (Fig 2M).

Hind margin of tergites and sternites IX–XI smooth. Dorsal lobe of segment XII with simple median pore, hind margin smooth. Ventral lobe of segment XII with about 10 teeth on hind margin (Fig 2E) and with 1+1 sternal anterolateral pores.

Female squama genitalis with pointed acrostyli (Fig 1G). Male squama genitalis with 6+6 setae, additional setae absent (Fig 2N).

***Body measurements*** *(based on 19 adults*, *in μm)*: Maximum body length 1780, head 180‒195, pseudoculus 11–12, posterior part of maxillary gland 17–19; hind cephalic setae *d7* 20–21, seta *sd7* 25–26, pronotal seta *1* 56–60, pronotal seta *2* 26–30, mesonotal setae *P1* 44–48, *P1a* 50–58, *P2* 70–73, *P2a* 15–16, foretarsus 120–125, claw 40–42, empodial appendage 5–7.

***Chaetal variability*:** Single specimens varied as follows: sternite III with 5 *A*-setae—symmetrical absence of seta *A1* (on 2 specimens), sternite III with duplication of seta *Ac* (1 specimen), sternite III with asymmetrical absence of seta *A1* (3 specimens), sternite VII with *Pc* absent (1 specimen).

***Type material and deposition*:** Holotype female (slide no. 77.1) from sample collected in litter and soil, steep southwest slope with *Quercus pubescens* over platy marl, Leopoldsberg, 48°16'40'' N, 16°20'37'' E, Vienna, Austria, 13.March.2012, coll. N. Szucsich and C. Resch.

Paratypes: 16 female and 2 male paratypes (no. 77.2–77.7, no. 6619–6625, HP 100–HP 103, HP 106, HP 107), and other material: 12 preimagos, 1 maturus junior (no. 6626–6638), collected together with the holotype. The holotype, 5 female and 1 male paratypes (slides no. 77.1–77.7) are deposited in the collection of the State Museum of Natural History of the National Academy of Sciences of Ukraine, L’viv (SMNH). Five female paratypes are deposited in the Museum of Natural History Vienna, Austria. Six females and one male paratypes (slides no. 6619–6625) and other material (12 preimagos and 1 maturus junior) are deposited in the collection of the Institute of Systematics and Evolution of Animals of the Polish Academy of Sciences, Kraków (ISEA).

***Distribution*:** Austria, known so far only from the type locality.

***Etymology*:** We have the honour of dedicating the new species to Dr. Erhard Christian, Vienna, in appreciation of his merits in studying the apterygote fauna of Austria.

***Remarks*:** *Acerentomon christiani* sp. nov. is similar to the group of species characterized by a short foretarsal sensillum *b*, the apex of which does not reach the base of *γ3*, and a broadened sensillum *a* (*A*. *gallicum*, *A*. *tenuisetosum*, *A*. *brevisetosum*, *A*. *italicum*, *A*. *fageticola* and *A*. *nemorale*). Furthermore, *A*. *christiani* differs from all *Acerentomon* spp. of the“*doderoi*” group in possessing a very long foretarsal sensillum *c*, which is longer than sensillum *a* and three times longer than *b*. In the remaining species sensillum *c* is shorter than *a* and about 1.5 times longer than sensillum *b*. The chaetotaxic pattern of the new species is most similar to *A*. *gallicum*, *A*. *tenuisetosum* and *A*. *brevisetosum*. However, *Acerentomon italicum*, *A*. *fageticola* and *A*. *nemorale* differ in seta *Pc* being absent from tergite VII, in the shape of the comb with lower number of teeth and in the length of the foretarsi (see the identification key). *Acerentomon christiani* sp. nov. is closest to *A*. *gallicum* in foretarsus and body lengths, and in the shape of laterotergal lines (lines on tergites II–V smooth, line of tergite VII with about 15 teeth and line on tergite VII with one or two strong teeth). These two species clearly differ in the length and position of sensillum *e* on the foretarsus (in the new species *e* is shorter and located at half the length between the bases of *d* and *f*, in *A*. *gallicum* this sensillum is long and very close to the base of sensillum *d*), in the shape of the comb (which possesses 12–14 long teeth in *A*. *gallicum*), and in the relative length ratio of mesonotal setae *P1* and *P1a* (in *A*. *christiani* sp. nov. the seta *P1a* is longer than seta *P1*, whereas in *A*. *gallicum P1a* is shorter). The position of the foretarsal sensillum *e* of the new species is similar to *A*. *tenuisetosum*, but the new species differs in possessing longer foretarsus and rostral setae and in the shape of the comb (see identification key below). *Acerentomon christiani* sp. nov. is closest to *A*. *brevisetosum* in the relative length ratio of mesonotal setae (*P1a* longer than *P1*), but differs in the length of the foretarsus and dorsal setae, in the shape of the anterolateral lines on tergites VI and VII, and in the shape of the comb (*A*. *brevisetosum* is characterized by shorter foretarsal length, very short dorsal setae of approximately 20 μm, smooth anterolateral lines on tergites VI and VII and possession of about 13 teeth on the comb).

#### Molecular description

**Fig 3:**
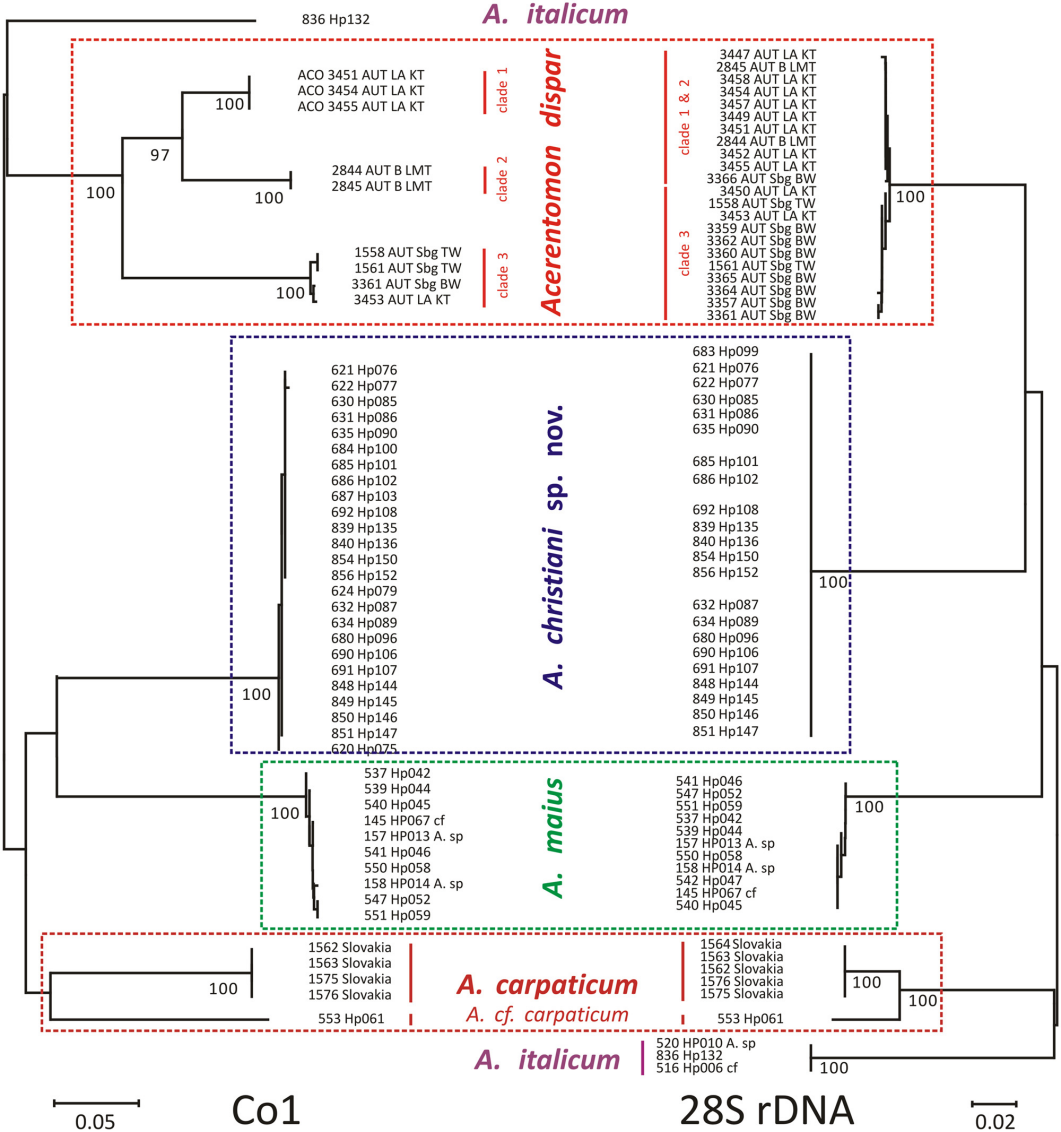
NJ tree based on K2P distances from 49 COI sequences and 65 28S rDNA sequences of *Acerentomon* spp. Bootstrap support (given below nodes) derived from 1000 replicates.

The DNA barcode of *Acerentomon christiani* sp. nov. is clearly delimited from all other *Acerentomon* species sequenced so far. The new species clusters with either *Acerentomon maius* (Fig 3, COI), or *Acerentomon dispar* (Fig 3, 28S).

## Discussion

The monophyly of the *Acerentomon* “*doderoi*” group is supported by the presence of supplementary seta *x* on tergite VII. Two other characters were briefly discussed by [15] as additional support: a distinctly protruded rostrum (LR 2.8 to 4.5) and the presence of 6 setae on tergite XI. Within the *doderoi* group only *A*. *novaki* has a short rostrum (LR 14). The presence of seta *P1a* on sternite VIII mentioned by [7] and [10] as an important character for distinguishing their *doderoi* group, was not confirmed by [15], who added species with only four setae on sternite VIII (*A*. *franzi* and *A*. *noseki*) into the group. Within the group only *A*. *franzi* is mentioned as having just four setae on tergite XI in the original description of [16]. However, in two male specimens from Vienna tergite XI has 6 setae. Two species (*A*. *skuhravyi* and *A*. *granulatum*) form a small subgroup, characterized by 6 setae on sternite IX and by an anterior position of setae *P1a* on sternite VIII ([17], [18]). All other chaetotaxic characters are uniform among all species of the group, with the exception of the chaetotaxy of segment VII. The presence or absence of seta *Pc* on tergite VII and sternite VII, and the absence of seta *A1* and presence of setae *P3a* on tergite VII, are of limited phylogenetic value, since [9] noted interspecific variability in these characters. The number of anterior setae on sternites I and III varies from 5 to 7 in different species, but this variation can be intraspecific, as observed both in the new species and mentioned by [9]. Species of the *Acerentomon doderoi* group clearly differ in length of sensillum *b*, shape of sensilla *a* and *b*, position of sensillum *e* on the foretarsus, shape of comb and in foretarsal length. The new species differs from all others by a very long foretarsal sensillum *c*, which reaches the base of the sensillum *t3* and is about three times longer than sensillum *b* (see identification key).

Both the tree based on the COI barcoding fragment and the tree based on the 28S rDNA fragment are fully congruent with morphological systematics. Since *A*. *christiani* sp. nov. is known only from a single locality little can be said with respect to its intraspecific variation. Distances are large among populations of *Acerentomon dispar*, sampled from four different locations in Austria (Fig 3, COI), a result congruent with [2]. However, intraspecific distances in the same species are nearly lacking in 28S rDNA sequences (Fig 3, 28S). A more conclusive contribution of molecular data to relationships among *Acerentomon* species awaits a denser sampling at both population- and species-level.

### Identification Key for All Described Species of the *Acerentomon “doderoi”* Group

1. Foretarsal sensillum *b* broad, slightly shorter to longer than sensillum *c* … 2

–Sensillum *b* slender and short, distinctly shorter than *c* … 10

2. Sensillum *b* longer than *c*, sensillum *a* broadened … 3

–Sensillum *b* slightly shorter than *c* … 6

3. Sternite VII with seta *Pc*, rostrum long… 4

–Sternite VII without seta *Pc* … 5

4. Sensillum *b* distinctly longer than c, its apex reaching base of seta *γ4*, length of foretarsus about 135 μm … *A*. *imadatei* Nosek, 1967 (Hungary, Slovakia, Austria)

–Sensillum *b* slightly longer than *c*, its apex not reaching base of seta *γ4*, length of foretarsus 154–160 μm … *A*. *baldense* Torti, 1986 (Italy)

5. Rostrum short (LR 14), sternite VIII with 6 setae, length of foretarsus about 105 μm… *A*. *novaki* Rusek, 1965 (Czech Republic)

–Rostrum of moderate length (LR 3.8), sternite VIII with 4 setae, length of foretarsus about 135 μm … *A*. *franzi* Nosek, 1965 (Austria)

6. Sensillum *a* broadened … 7

–Sensillum *a* slender … 8

7. Tergite VII with 18 posterior setae, comb with about 60 teeth, LR 3.2 … *A*. *rostratum* Ionesco, 1951 (Romania)

–Tergite VII with 16 posterior setae, comb with 14–16 teeth, LR 2.6–2.7 … *A*. *maius* Berlese, 1908 (Italy, Slovakia, Slovenia, Austria)

8. Setae *P1a* on sternite VIII placed proximally, close to *P1* and *P2*, sternite IX with 6 setae, rostrum long (LR 2.7)… 9

–Setae *P1a* on sternite VIII placed distally, on hind margin of sternite, sternite IX with 4 setae, rostrum of medium size (LR 3.7) … *A*. *doderoi* Silvestri, 1907 (Italy, Slovenia, other records are questionable)

9. Comb with about 25 teeth, length of foretarsus about 125 μm *… A*. *skuhravyi* Rusek,1965 (Slovakia, Poland, Ukraine)

–Comb with about 40 teeth, length of foretarsus about 140 μm … *A*. *granulatum* Szeptycki, 1993 (Georgia)

10. Foretarsal sensillum *b* reaching base of seta *γ3* … 11

–Foretarsal sensillum *b* not reaching base of seta *γ3*… 15

11. Sensillum *a* broadened, sword-shaped … 12

*–*Sensillum *a* slender … 14

12. Tergite VII and sternite VII with seta *Pc* Tergite VII with 19 posterior setae, LR 3.3, length of foretarsus about 150 μm … *A*. *dispar* Stach, 1954 (Poland, Czech Republic, Slovakia, Ukraine, Austria)

*–*Tergite VII and sternite VII without seta *Pc* … 13

13. Tergite VII with 14 posterior setae …

*A*. *kustorae* Nosek, 1983 (Slovenia)

*–*Tergite VII with 16 posterior setae (for additional characters see current redescription [19]) … *A*. *italicum* Nosek, 1969 (Italy, Austria)

14. Sternite VII with *Pc*, length of foretarsus 156–164 μm, rostrum long (LR 3.1) … *A*. *tuxeni* Nosek, 1961 (Slovakia, Austria, Czech Republic, Poland)

*–*Sternite VII without *Pc*, length of foretarsus about 145 μm, rostrum long (LR 2.8) … *A*. *giganteum* Condé, 1944 (Austria, Czech Republic, France, Germany, Poland, Slovakia, Slovenia, Africa)

15. Tergite VII without seta *Pc* … 16

*–*Tergite VII with seta *Pc* …19

16. Tergite VII with 12 anterior setae (*A1* present) … 17

*–*Tergite VII with 10 anterior setae (*A1* absent) … *A*. *nemorale* Womersley, 1927 (Great Britain, France, Luxembourg, Germany, Austria, Slovakia, Czech Republic)

17. Sternite VII with seta *Pc*, length of foretarsus about 135 μm …. 18

*–*Sternite VII without seta *Pc*, length of foretarsus 105–122 … *A*. *fageticola* Rusek, 1966 (Czech Republic, Slovakia, Poland, Austria)

18. Sternites VIII and XI with 4 setae, comb with about 32 teeth, rostrum long (LR 3.3) … *A*. *noseki* Torti, 1981 (Italy)

*–*Sternites VIII and XI with 6 setae, comb with 10‒16 teeth, rostrum of medium size (LR 4.0–4.5) … *A*. *omissum* Szeptycki, 1980 (Poland, Slovakia, Ukraine)

19. Length of foretarsus about 110 μm … 20

*–*Length of foretarsus about 125 μm … 21

20. All dorsal setae very short, comb with about 13 teeth, rostrum long (LR 3.0–3.2)… *A*. *brevisetosum* Condé, 1945 (France)

*–*All dorsal setae long, comb with about 8 teeth, rostrum of moderate length (LR 3.8) … *A*. *tenuisetosum* Nosek, 1973 (Great Britain)

21. Sensillum *a* longer than *c*, comb with 12–14 teeth, mesonotal seta *P1a* shorter than *P1* … *A*. *gallicum* Ionesco, 1933 (France, Austria, Czech Republic, Germany, Italy, Slovakia, Poland)

*–*Sensillum *a* shorter than *c*, comb with 18–22 teeth, mesonotal seta *P1a* longer than *P1 … A*. *christiani* sp. nov. Shrubovych & Resch (Austria)
